# Treatment seeking for alcohol-related issues during the COVID-19 pandemic: An analysis of an addiction-specialized psychiatric treatment facility

**DOI:** 10.1016/j.heliyon.2022.e09934

**Published:** 2022-07-14

**Authors:** Mitchell J. Andersson, Anders Håkansson

**Affiliations:** a Department of Clinical Sciences Lund, Faculty of Medicine, Lund University, Sölvegatan 19 – BMC F12, 221 84 Lund, Sweden; b Malmö Addiction Center, Clinical Research Unit, Skåne Region, Södra Tullgatan 4, 211 40 Malmö, Sweden

**Keywords:** Alcohol use, Interrupted time series analysis, Alcohol consumption, Alcohol use disorder, Treatment uptake, Substance use disorder

## Abstract

The COVID-19 pandemic and its societal impact may cause long-term behavioral changes in alcohol use due to increased psychological distress, unemployment, and time spent home. The purpose of this study was to determine whether or not the COVID-19 pandemic had a significant impact on treatment seeking for alcohol use disorders and related problems in a Swedish psychiatric treatment facility. Using an interrupted-time-series design on data derived from an addiction-specific psychiatric treatment facility located in Malmö, Sweden, we hypothesized that treatment seeking would decrease during the pandemic based on previous research identifying limited alcohol availability and affordability, as well as accessibility to treatment centers as influential factors. In addition, we assessed the predictive power of alcohol sales and number of active cases in the region using simple linear regressions. Results indicated that the pandemic had little to no effect on the number of people needing care, however a significant step change was found in treatment seeking patterns for unique female patients during the second wave (October 2020). Regression analyses indicated that alcohol sales and the number of active cases in the region did not significantly predict treatment seeking. A causal relationship between the onset of the pandemic and variation in treatment seeking for alcohol use could not be established. More research is needed to fully understand the pandemic’s impact on alcohol use behavior change.

## 1 Introduction

The spread of the coronavirus disease 2019 (COVID-19) pandemic continues to pose a serious threat to the public’s physical and mental health. The challenges and changes in daily life stemming from the pandemic has likely had a profound effect on the population's mental health, possibly contributing to a rise in psychological distress and substance use (Czeisler et al., 2020; Marsden et al., 2020; McGinty et al., 2020; Stanton et al., 2020). Because of its prevalence and abuse liability, alcohol is a drug of particular concern.

Prior research demonstrates that trends for alcohol consumption during crises similar to the COVID-19 pandemic are not unidirectional, with both increases and declines being recorded (de Goeij et al., 2015; Rehm et al., 2020). Higher rates of alcohol consumption are linked to coping with feelings of isolation, distress, uncertainty about the future, and general decline in mental health, while decreases in alcohol consumption are often linked to a decrease in availability and affordability of alcohol due to policy changes and higher unemployment rates (Clay and Parker, 2020; Columb et al., 2020; Rehm et al., 2020). The COVID-19 crisis involves a complex interplay between multiple factors that influence the rise and fall of alcohol consumption. Many countries understood the importance of restricting the selling of alcohol during the early stages of the pandemic and faced the problem of enforcing restrictions or even banning its sale. Recent studies conducted in countries where a temporary alcohol ban was enforced, such as South Africa and India, reported both positive and negative effects as a result. For example, the total number of trauma cases in one South African hospital's emergency department dropped by 47% after the ban (Morris et al., 2020), while the number of patients admitted with alcohol withdrawal syndrome in a number of Indian hospitals doubled (Narasimha et al., 2020). In a Scandinavian context where limitations were implemented early on, a study conducted by Mäkelä et al. (2021) discovered no change in alcohol consumptions in Norway and a 9% decrease in drinking in Finland. The Swedish Health Authorities' initial reaction was steadfast in contrast; no prohibitions or extreme limits on the sale of alcohol were immediately enforced (Keane and Neal, 2021). Few studies have examined the consequences of these early decisions in the Swedish context, but a recent cross-sectional study by Kilian et al. (2021) found that since the outbreak of the pandemic, most European countries, including Sweden, have seen a decrease in self-reported alcohol consumption, with several Swedish news outlets reporting similar patterns. Another recent study comparing the drinking habits of Swedish adult workers during the first (April–June) and second (October–December) waves of the pandemic found a small decrease in the number of participants who self-reported a negative change in drinking habits during the second wave compared to the first wave (Blom et al., 2021). Furthermore, several media sources in Sweden reported that since the limiting of bar and restaurant serving hours during the second wave of cases, the number of calls to emergency services to report violent crime in the evening hours has decreased significantly (Liljesson, 2021). This could be another indicator that alcohol intake has declined because increased alcohol consumption is positively correlated to an increased risk of interpersonal conflict and, as a result, violent behavior (Brecklin, 2002; Leonard and Quigley, 1999; Testa et al., 2003).

Despite the apparent decrease in alcohol consumption, the need for psychiatric care for alcohol-related problems and disorders is unlikely to have declined since the start of the pandemic (Yang et al., 2020). As Narasimha and colleagues' (2020) study in India demonstrated, a decrease in alcohol use among dependent individuals may result in increased treatment seeking among those suffering from alcohol withdrawal syndrome. Despite this, perceived barriers to treatment seeking may dissuade individuals from scheduling initial visits or impede those with pre-existing alcohol use disorders from scheduling follow-up visits, particularly among individuals seeking treatment to reduce their drinking. Tighter travel restrictions and the risk of exposure to the virus at psychiatric treatment facilities are major barriers that treatment seekers face in the current crisis (Håkansson et al., 2021). A recent study conducted in the United States found that these barriers are especially challenging for those with single- and polysubstance use disorders in need of more intensive in-person care and treatments incorporating group therapy (Mellis et al., 2021). Similar fears have been expressed for individuals with other non-alcohol related addictions (Glowacki et al., 2021; Yang et al., 2020). Moreover, it is unclear how the pandemic has impacted various age groups and individuals that drank more before the pandemic. For example, some studies have highlighted that older generations endorsed better well-being ratings during the first month of the pandemic compared to five years prior to the pandemic's onset (Kivi et al., 2021). A similar study measuring life satisfaction found a greater proportion of the Swedish population expressing contentment or improvement with their life during the months of the pandemic (Brogårdh et al., 2021). Concerning alcohol consumption, the virus' impact on groups already struggling with or on the verge of alcoholism is particularly concerning (Finlay and Gilmore, 2020; Kim et al., 2020). Heavy drinkers, for example, may increase their drinking at home to compensate for their inability to drink elsewhere (Callinan et al., 2021). Beyond alcohol consumption, several intrapersonal factors, such as an individual's personality, level of psychological distress, impulsivity, aggression, mood impairments, and liver enzyme abnormalities, may all contribute to a greater likelihood alcohol-dependent persons seek treatment (Rohn et al., 2017). Psychological and physiological differences between individuals may interact with changes in daily life brought about by the pandemic and influence treatment seeking behavior. Therefore, there is a critical need for research into how pandemic-related changes have impacted the uptake of treatment for alcohol misuse and alcohol use disorders.

Based on recent research and media reports indicating a decrease in alcohol consumption and general treatment seeking during COVID-19, the present study hypothesized that there would be a decrease in treatment seeking for alcohol-attributable issues after the onset of the pandemic. In addition, we hypothesized that alcohol sales and number of COVID-19 cases in the region would have an effect on the number of patients seeking treatment. Using data obtained from a specialized treatment unit in southern Sweden, the aim of the study was to determine the monthly variations in alcohol-attributable admissions both preceding and during the COVID-19 pandemic as well as whether related factors contributed to changes in admission-rate. Data from the two years prior to the outbreak served as a historic baseline for comparison.

## 2 Materials and methods

Treatment seeking data were collected from an addiction-specific psychiatric treatment facility located in Malmö, Sweden. The facility is run by Region Skåne, the healthcare provider for the 1.3 million residents of Skåne, Sweden’s southernmost county. The treatment center admits patients over the age of 18 with alcohol use disorders and related problems from the urban district of Malmö and neighboring municipalities and provides them with publicly tax funded-medical psychiatric assessment and therapy. This is a unit especially geared toward patients who do not require formal collaboration with social authorities, which is necessary in cases when the individual’s drinking has a significant impact on their social life by means of, for example, housing issues and major financial problems. As a result, the current unit is more suited to this type of research, where we are interested in true treatment seeking, rather than initiatives taken by other authorities in the form of, for example, compulsory care. An important note to address about the facility is, because of an annual general drop in staff coverage between late June and early August due to summer holidays, there is often some degree of seasonality in treatment uptake at the facility during the summer months. Nevertheless, services were not suspended at any point during the crisis and video-based treatment was made available for patients.

March 2020 is considered the point when the COVID-19 pandemic first had a significant impact on Swedish society. Due to a spike in cases during this time and the World Health Organization’s (WHO) declaration of COVID-19 as a pandemic (WHO, 2020), the government imposed a number of directives on the public, such as work-from-home orders on March 16th and the closure of high school and university campuses on March 17th. Although the rest of Swedish society remained essentially open during this time, Sweden introduced a travel ban to the rest of the European Union on March 17th, which prevented many Swedes from traveling to Denmark and Germany to buy large quantities of alcohol at lower prices. On March 29th, gatherings of fifty or more people were prohibited, and social distancing recommendations took effect on April 1st. More restrictions, such as restrictions on serving hours for restaurants and bars, were introduced during subsequent waves to control the spread of the virus (Socialdepartementet, 2020a). The Swedish COVID-19 legislation (Swedish COVID-19 Law, SFS 2021:4) and vaccine protocols (Socialdepartementet, 2020b) took effect in early January, 2021, and restrictions were not lifted until September 29th, 2021 (Kulturdepartementet and Socialdepartementet, 2021), which is outside of the window of the present dataset.

### 2.1 Measures

Treatment seeking data for alcohol-related issues were extracted from the regional healthcare databases. We collected the number of unique male (*n* = 2535_*males*_) and female patient contacts (*n* = 1495_*females*_; *n* = 4030_*total—unique*_; defined as a patient seeking treatment for alcohol attributable issues for the first time at the facility) plus the total number of patient contacts per month (*n* = 5908_*total—contacts*_; defined as the total number of patient contacts related to alcohol attributable issues with medical personnel at the facility, including initial visit, meaning each patient may be responsible for multiple contacts) for alcohol-related issues between January 2018 and June 2021 (*n* = 42_*months*_). Data did not contain any personally identifiable information, such as pre-existing conditions or specific medical diagnoses, and age and sex were reported on a group level. Ethical approval for the study was applied for jointly by the second author (AH) and the director of Malmö Addiction Center who is the officer responsible of the health care data used in the present study, and who is responsible of providing access to this register data. The ethics committee judged the project not to require formal ethical approval, as it does not involve identified individual data (Swedish Ethical Review Authority, decision number 2020–03232, August 19, 2020).

In Sweden, alcoholic beverages with an alcohol content greater than 3.5 percent must be purchased through the state-owned retail monopoly, Systembolaget. Alcohol sales in 1000’s of liters through Systembolaget from quarter one of 2017 to quarter two of 2021 were extracted from quarterly reports released on their public website (https://www.omsystembolaget.se). Cumulative liters of sold liquors, wines, beers, ciders, and assorted drinks were summed for each quarter. Sales of alcohol-free beverages were not included. Concerning the number of coronavirus cases in Skåne, data were extracted from Region Skåne’s publicly published situation reports for daily confirmed COVID-19 cases in Skåne, Sweden that is updated daily (https://www.skane.se/digitala-rapporter/lagesbild-covid-19-i-skane).

### 2.2 Statistical methods

Monthly patient admission data were analyzed using interrupted-time-series (ITS) analyses to assess the longitudinal effects of the COVID-19 pandemic on each of the treatment seeking outcome variables (total contacts, unique patients, unique female patients, and unique male patients). Since the COVID-19 pandemic first impacted Swedish society in early March 2020, data were divided into three time periods: the initial wave of COVID-19 in March 2020 (Wave 1), the emergence of the second wave of cases in October 2020 (Wave 2), and non-COVID-19 affected months (pre-COVID-19), with March 2020 and October 2020 used as interruption points. For the ITS analysis, data were log transformed in order to be later interpreted as percentages. Autoregressive integrated moving average (ARIMA) models were fitted to the data using the *auto.arima()* function of the *forecast* package in R (R Core Team, 2016). We employed the studentized Breusch-Pagan test to determine heteroscedasticity (Breusch and Pagan, 1979), and the Augmented Dickey-Fuller (Dickey and Fuller, 1979) and Ljung-Box (Ljung and Box, 1978) Tests to determine stationarity and the presence of autocorrelation, as recommended by Schaffer et al. (2021). Models were selected based on the satisfaction of assumptions and lowest Akaike information criterion (AIC). The process of model testing is presented in supplemental Table 1.

Alcohol sales measured by 1000s of liters are reported quarterly by the government sponsored alcohol dispenser (Systembolaget, 2021). Cubic-spline interpolation was used using MATLAB R2020b to convert data to monthly values. In order to simplify the interpretation of regression coefficients, they were further divided by 1000 and measured in millions of liters sold per month. Likewise, daily confirmed cases reported by Region Skåne were aggregated into monthly values, which served as an index for the prevalence of the virus and perceived barrier for treatment seeking. Because of the changes cited regarding alcohol sales and the number of COVID-19 cases over time, we conducted linear regressions to assess the relationship these variables have with treatment seeking outcomes. Linear regressions were conducted using SPSS 27.0 for Windows.

## 3 Results

**Figure 1:**
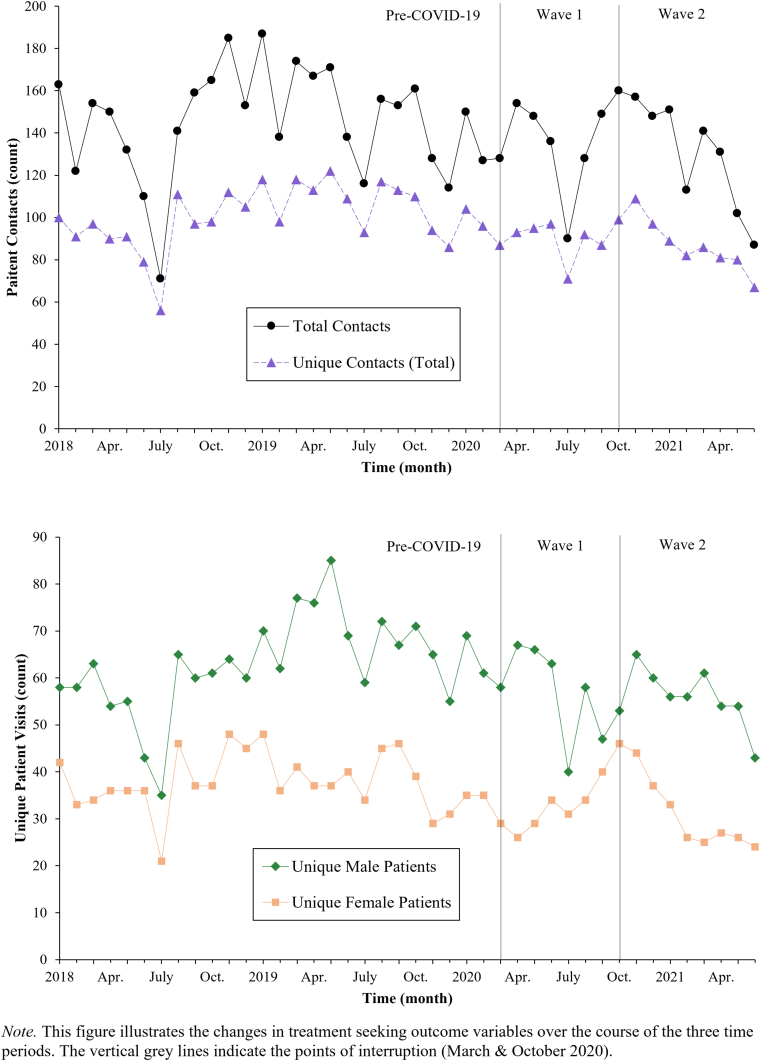
Alcohol-Attributable Patient Contacts per Month (January 2018–June 2021).

Overall, there were no significant trend changes in the total number of patient contacts (*M*_*pre*_ = 145.58, *SD* = 25.95 vs. *M*_*post*_ = 132.69, *SD* = 23.42), unique patients (*M*_*pre*_ = 100.69 *SD* = 14.42 vs. *M*_*post*_ = 88.25, *SD* = 10.64), unique male patients (*M*_*pre*_ = 62.85, *SD* = 10.25 vs. *M*_*post*_ = 56.31, *SD* = 7.84), nor unique female patients (*M*_*pre*_ = 37.85, *SD* = 6.23 vs. *M*_*post*_ = 31.94, *SD* = 6.85) at the facility before versus during the pandemic when taking secular trends into consideration, as illustrated in Figure 1. However, a significant step change was found among unique female patients following the October 2020 interruption, +41.7%, 95% CI [12.3,71.4], *z* = 2.49, *p* = .01. Temporary step and long-term trend changes are presented as percentages in Table 1.

**Table 1:** Estimated relative changes in treatment seeking at each point of interruption.

Variable	Monthly *M* before March 2020 (#)	Temporary change March 2020 (%)	Temporary change: October 2020 (%)	Trend change: Apr 2020–June 2021 (%)
Total Contacts	145.6	−19.2 [−46.5,8.0]^a^	7.7 [−23.6,39.0]^a^	−1.1 [−8.4,6.3]
Total Unique Patients	100.7	−5.2 [−29.0,18.7]^b^	25.3 [0.3,50.4]^b^	−1.3 [−4.1,1.5]
Unique Males	62.9	−2.2 [−30.4,25.9]^b^	18.1 [−12.6,48.8]^b^	−1.4 [−4.7,1.9]
Unique Females	37.9	−14.9 [−51.3,21.4]^b^	41.7∗ [12.3,71.4]^b^	−1.0 [−6.2,4.2]

*Note.* Analyses conducted using ARIMA modeling with points of interruption set to March 2020 (Wave 1) and October (Wave 2).

a Fitted using SARIMA (0,1,0) (0,1,1) model.

b Fitted using ARIMA (0,1,1) model.

∗ *p* < .05.

**Figure 2:**
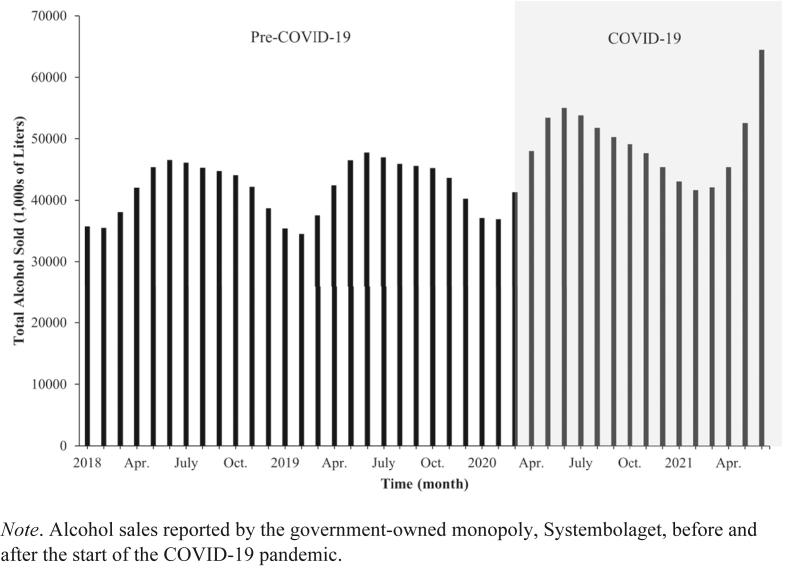
Total Alcohol Sales via Systembolaget per Month (January 2018–June 2021).

A series of simple linear regressions were run to determine the strength of alcohol sales (Model 1; Figure 2) and number of COVID-19 cases in Skåne (Model 2) as independent predictors of treatment seeking during the pandemic time period (March 2020–June 2021). Assumptions pertaining to linearity, independence of residuals, multicollinearity, and normality were met according to satisfactory partial regression plots, Durbin-Watson statistics, tolerance values, Cook’s distances, and Q-Q plots. Regression results for Model 1 found that alcohol sales did not significantly predict total contacts, *F*(1,15) = 3.96, *p* = .07; total unique patients, *F*(1,15) = 1.68, *p* = .22; unique male patients, *F*(1,15) = 2.62, *p* = .13; nor unique female patients, *F*(1,15) = 0.04, *p* = .84. Likewise, regression results for Model 2 found that the number of COVID-19 cases in Skåne did not significantly predict total contacts, *F*(1,15) = 1.00, *p* = .34; total unique patients, *F*(1,15) = 0.21, *p* = .39; unique male patients, *F*(1,15) = 0.48, *p* = .50; nor unique female patients, *F*(1,15) = .334, *p* = .57. Regression coefficients, standard errors, and model multiple correlation squared (*R*^2^) values can be found in Table 2.

**Table 2:** Linear regression results for treatment seeking outcome variables.

Variable	Model 1			Model 2		
	*B*	*β*	*SE*	*B*	*β*	*SE*
Total Contacts						
Constant	132.69∗∗		5.35	132.69∗∗		5.86
Alcohol Sales	−1.79	−.47	0.90			
COVID-19 Cases				.51	.26	0.51
*R*^2^	.22			.07		
Total Unique Patients						

Constant	88.25∗∗		2.60	88.25∗∗		2.68
Alcohol Sales	−0.57	−.33	0.44			
COVID-19 Cases				.21	.23	0.23
*R*^2^	.11			.05		
Unique Male Patients						

Constant	56.31∗∗		1.86	56.31∗∗		2.00
Alcohol Sales	−0.51	−.40	0.31			
COVID-19 Cases				.12	.18	0.17
*R*^2^	.16			.03		
Unique Female Patients						

Constant	31.94∗∗		1.77	31.94∗∗		1.75
Alcohol Sales	−0.06	−.05	0.30			
COVID-19 Cases				.09	.15	0.15
*R*^2^	.003			.02		

*Note*. Alcohol Sales and COVID-19 Cases variables were presented in millions and thousands, respectively, and centered. In Model 1, we entered Alcohol Sales (1,000,000 s of liters) to predict treatment seeking outcome variables. In Model 2, we entered COVID-19 Cases in Skåne (1,000 s) to predict treatment seeking outcome variables. Model = “Enter” method in SPSS; *B* = unstandardized regression coefficient; CI = confidence interval; *LL* = lower limit, *UL* = upper limit; *SE* = standard error of unstandardized coefficient; β = standardized coefficient; *R*^2^ = coefficient of determination.

∗*p* < .05. ∗∗*p* < .01.

## 4 Discussion

The present study aimed to compare the number of alcohol-attributable admissions at an addiction-specialized treatment facility prior to versus during the COVID-19 pandemic. This investigation contributes to the understanding of how the crisis has impacted treatment seeking for alcohol misuse and alcohol use disorders in Sweden, which has been widely discussed among researchers and in the media. Additionally, since Sweden's response approach differed from that of many other countries, the current study serves as a benchmark to which future studies can be compared. More research is needed to compare how a non-lockdown approach affects alcohol use and treatment seeking relative to an environment where temporary lockdown steps are in effect.

We hypothesized that alcohol-attributable contacts would decline during the months following the onset of the pandemic due to a number of factors, including the limited availability and affordability of alcohol (Clay and Parker, 2020; Columb et al., 2020; Rehm et al., 2020), an increased number of perceived barriers to treatment seeking (Håkansson et al., 2021), and earlier studies citing a decline in alcohol consumption after the outbreak (Kilian et al., 2021). The results largely did not support this hypothesis, as differences were minor or inconclusive across treatment seeking outcome variables. However, there was a significant temporary change in female treatment seeking in the positive direction occurring in October 2020 at the start of the second wave of the pandemic. In addition, we hypothesized that alcohol sales and COVID-19 cases in the region would predict treatment seeking patterns during the pandemic and the results did not support this hypothesis. Over the duration of the pandemic, neither alcohol sales nor number of COVID-19 cases significantly predicted treatment seeking.

Provided that the overall number of alcohol-related admissions was unaffected, this suggests that the decrease in alcohol use and increase in alcohol sales through Sweden's alcohol retailer are unrelated to treatment seeking, as evidenced by our linear regression models which did not identify a significant correlation between alcohol sales and treatment seeking outcome variables (Kilian et al., 2021, Figure 2; Table 1). However, the lack of a relationship could also be explained by delayed effects or inter-group treatment seeking differences. In other words, changes in drinking behavior associated with the pandemic may differ between the general population and populations already at risk of developing alcohol use disorders. For instance, a small decrease in treatment seeking among the general population would balance a substantial increase in treatment seeking among members from vulnerable groups. In the case of females seeking treatment for the first time, the temporary increase observed in October 2020 could be the result of a delayed effect, as problem drinking behaviors are more likely to develop in response to chronic stressors as opposed to acute stressors (Becker et al., 2011). A difference in treatment seeking patterns between sexes was not unexpected (Rodriguez et al., 2020), as earlier studies have shown that men are more likely to receive specialized treatment for alcohol use disorders than women, who are more often treated for mental illnesses (Edlund et al., 2012; Harris et al., 2016). However, the temporary change following the October 2020 interruption point was contrary to expectations, but similar to the findings of Blom et al. (2021), who discovered that women were less likely to reduce their alcohol intake following the outbreak. Studies measuring pandemic-related changes in mental health and well-being have found that women have been negatively affected to a greater degree than men (Etheridge and Spantig, 2020). More specifically, several studies in European populations cite a larger proportion of women having reported stronger feelings of loneliness and psychological distress (Bu et al., 2020; Petzold et al., 2020), which have been found to be associated with greater alcohol consumption long-term (Åkerlind and Hörnquist, 1992; Segrin et al., 2018).

Stress, both acute and chronic, has been shown to play a critical role in the initiation and continuation of alcohol abuse, as well as relapse (Brady and Sonne, 1999). In the context of a crisis, research has shown that economic stressors can lead to significant psychological distress and subsequent problem drinking behavior (de Goeij et al., 2015). In the Swedish context during COVID-19, the number of furloughs and the unemployment rates were highest amongst younger age groups (Campa et al., 2021) suggesting that this group was at an elevated risk for stress-induced problem drinking behavior. This was concerning, considering this group already accounts for the majority of patients seeking treatment for alcohol-related substance use disorders (Edlund et al., 2012). Acute stressors, as opposed to chronic stressors, are generally less impactful on drinking behavior (Becker et al., 2011), which may explain why there was not a steep rise in admissions during the early stages of the pandemic. However, it is unlikely that the general public could foresee how long the pandemic would continue to affect daily life. After the early stages of the pandemic, a greater prevalence of chronic stress and anxiety about the future may have intensified poor mental health to a greater degree among those in at-risk groups and those with pre-existing alcohol use disorders (Callinan et al., 2021), however, the results indicate little to no trend change in treatment seeking, which is encouraging to an extent.

Although the number of perceived barriers to treatment seeking likely increased overall with the number of COVID-19 cases in the area, the number of active cases did not predict treatment seeking. This effect, or lack thereof, may likewise be age-related. Since coronavirus and its variants have been shown to be significantly more dangerous in older populations (Hägg et al., 2020), who are not in the age-range for prototypical alcohol-related treatment seekers, the risk for infection and death was likely perceived to a lesser degree in the younger populations (Niño et al., 2021). Therefore, the barriers to treatment were likely less challenging to overcome for this group. Furthermore, studies in other European countries have found that those that were younger, female, more psychologically and financially distressed, and had premorbid anxiety disorders were more likely to increase their drinking during the pandemic (Garnett et al., 2021). Ultimately, younger female populations may have been more likely to increase their drinking than men and perceive fewer substantial barriers to treatment than older populations, which may account for the brief increase in female patients seeking treatment for the first time ahead of the second wave.

While the impact of the pandemic on treatment seeking was negligible overall, the positive step change among unique female seekers during the second wave of cases in October 2020 was unexpected for admissions at an addiction-specialized treatment facility. This could indicate that the pandemic's impact on women's mental health had resulted in greater drinking and a higher incidence of problem drinking temporarily. Overall trends remained stable, indicating that these temporary changes are an effect of the pandemic, rather than extraneous factors. In the future, psychiatric facilities may see an increase in female patients seeking treatment for alcohol-related issues and disorders months after a crisis that presents similar persistent stressors as the COVID-19 pandemic has. Given the likelihood of further waves of the pandemic occurring as a result of novel coronavirus variants, support systems and treatment facilities must be prepared for an abnormally high number of new patients throughout this time period while the highs and lows of the pandemic come and go.

### 4.1 Limitations

The present study is not without its limitations. First and foremost, drawing conclusions from a study conducted during a period when several complex factors are at play that could have a major effect on outcome variables is challenging. There are numerous factors other than the level of fear and psychological distress in the population that contributed to treatment seeking patterns. For example, macro factors such as the extent to which the crisis has been covered in the media and the number of government interventions implemented to contain its spread can have a direct or indirect effect on the relationships between alcohol consumption as a result of psychological distress and treatment seeking. In addition, significant intrapersonal covariates, such as history of mental illness, current physical condition, homelessness, marriage status were unable to be controlled for using univariate time series analyses. These variables have likely had an influence on how people perceive the feasibility of seeking treatment at the facility. Moreover, the study's findings on pandemic-related changes are not easily generalizable to other regions in Sweden and other nations that were affected to a lesser or greater degree than the county in which the present study was carried out (Skåne). The effects of the COVID-19 crisis in Sweden alone varied widely, as shown by the fact that the number of cases reported in Sweden's larger counties, such as Stockholm (2.4 million inhabitants) and Västra Götaland (1.7 million inhabitants), more than doubled those reported in Skåne. The results should be interpreted with caution since the present analysis did not directly measure psychological distress and alcohol consumption, nor was it able to control for longitudinal fluctuations using linear regression.

Since the data were obtained from a single specialized addiction unit, our sample size was limited, which resulted in a higher degree of statistical uncertainty. Other psychiatric treatment facilities around Sweden do not offer the same services as the regional clinic in Malmö, and therefore, a nationwide review could not be carried out. Furthermore, because we were unable to collect data specific to individual patients, it is possible that a single unique patient was counted in the data at multiple time points, resulting in figures from various months being unable to be summed. Therefore, the number of cumulative contacts, rather than the absolute number of new admissions, represents the treatment facility’s workload and general treatment uptake of the treatment facility for alcohol-related issues. Lastly, since only primary diagnosis data were available, it was impossible to determine whether there were longitudinal changes in alcohol-related problem severity or other aspects of care during the crisis. As a result, psychiatric comorbidity could not be investigated, however, comorbidities are less prevalent and less severe in this unit than others.

## 5 Conclusion

The purpose of the present study was to investigate whether the COVID-19 pandemic had a significant impact on alcohol-related treatment seeking in Sweden. Results indicated that the pandemic largely did not impact the number of individuals seeking treatment, but changes in treatment seeking patterns were observed among unique female patients between the first and second waves of COVID-19 cases in the area. Since the current study was unable to account for fluctuations in psychological distress and fear as a consequence of the pandemic, further research is required to fully comprehend the pandemic’s impact on alcohol-related treatment seeking in general and within at-risk groups. Considering the widespread fear of substance-related problems at the start of the pandemic, the lack of change in treatment seeking patterns is encouraging but does not eliminate the possibility that the pandemic had more significant effects on drinking behavior than suggested in the present study.

## Statements of ethical approval

The study was reviewed by the Swedish Ethical Review Board, which determined that the project did not require formal ethical approval because it did not include personally identifiable data (file number 2020–03232).

## Declarations

### Author contribution statement

Mitchell J. Andersson, MSc: Performed the experiments; Analyzed and interpreted the data; Contributed reagents, materials, analysis tools or data; Wrote the paper.

Anders Håkansson, PhD: Conceived and designed the experiments; Performed the experiments; Contributed reagents, materials, analysis tools or data; Wrote the paper.

### Funding statement

This research did not receive any specific grant from funding agencies in the public, commercial, or not-for-profit sectors.

### Data availability statement

Data included in article/supp. material/referenced in article.

### Declaration of interests statement

The authors declare the following conflict of interests:

The second author has previously received overall research funding from AB Svenska Spel, the state-owned gambling operator in Sweden.

The present study was supported by the second author's overall research funding from the regional health care system (Region Skåne).

Neither AB Svenska Spel nor Region Skåne had any role in the study design, collection, analysis or interpretation of the data, drafting of the manuscript, or the decision to submit this paper for publication.

### Additional information

Supplementary content related to this article has been published online at https://doi.org/10.1016/j.heliyon.2022.e09934.

## Appendix A Supplementary data

Supplementary_Materials_Andersson_Hakansson2021_V3 Supplementary_Materials_Andersson_Hakansson2021_V3
