# Supplementary material for: Treatment seeking for alcohol-related issues during the COVID-19 pandemic: An analysis of an addiction-specialized psychiatric treatment facility
Source: Heliyon. 2022 Jul 14;8(7):e09934. doi: 10.1016/j.heliyon.2022.e09934 (PMC9279183; doi:10.1016/j.heliyon.2022.e09934)
Supplement: Supplementary_Materials_Andersson_Hakansson2021_V3 [file mmc1.docx]

**Supplementary Materials**

**Table 1**

| *ARIMA Modeling Table for Treatment Seeking Outcome Variables* | | | | | | | |
| --- | --- | --- | --- | --- | --- | --- | --- |
| ARIMA Model | Breusch-Pagan | | Dickey-Fuller | |  | Ljung-Box | |
|  | BP | *p* | DF | *p* | AIC | Q* | *p* |
| Total Contacts | | | | | | | |
| March 2020 |  |  |  |  |  |  |  |
| (0,0,0) | 0.42 | .52 | -2.57 | .35 |  |  |  |
| (0,1,0) |  |  | -4.99 | < .01 | -0.94 | 24.37 | < .001 |
| (0,1,0) (0,1,0) |  |  |  |  | -15.21 | 5.21 | .52 |
| (0,1,0) (1,1,0) |  |  |  |  | -21.82 | 5.03 | .41 |
| (0,1,0) (0,1,1)^a^ |  |  |  |  | -22.69 | 5.26 | .38 |
| October 2020 |  |  |  |  |  |  |  |
| (0,1,0) |  |  |  |  | -1.79 | 26.07 | < .001 |
| (0,1,0) (0,1,0) |  |  |  |  | -12.7 | 7.84 | .25 |
| (0,1,0) (1,1,0) |  |  |  |  | -19.9 | 6.35 | .27 |
| (0,1,1) (1,1,0) |  |  |  |  | -21.0 | 4.95 | .29 |
| (0,1,0) (0,1,1)^a^ |  |  |  |  | -20.8 | 6.64 | .25 |
| Total Unique Patients | | | | | | | |
| March 2020 |  |  |  |  |  |  |  |
| (0,0,0) | 1.07 | .30 | -1.69 | .69 |  |  |  |
| (0,1,0) |  |  | -4.61 | < .01 | -23.01 | 25.52 | < .001 |
| (0,1,1)^a^ |  |  |  |  | -34.20 | 5.61 | .35 |
| (0,1,0) (0,1,0) |  |  |  |  | -20.87 | 3.67 | .72 |
| (0,1,0) (0,1,1) |  |  |  |  | -26.08 | 3.76 | .58 |
| October 2020 |  |  |  |  |  |  |  |
| (0,1,0) |  |  |  |  | -23.76 | 25.08 | < .001 |
| (0,1,1)^a^ |  |  |  |  | -37.82 | 5.66 | .34 |
| (0,1,0) (0,1,0) |  |  |  |  | -18.39 | 11.64 | .07 |
| (0,1,0) (1,1,0) |  |  |  |  | -24.85 | 8.86 | .11 |
| (0,1,0) (0,1,1) |  |  |  |  | -25.26 | 8.51 | .12 |
| Unique Male Patients | | | | | | | |
| March 2020 |  |  |  |  |  |  |  |
| (0,0,0) | 0.85 | .36 | -1.98 | .58 |  |  |  |
| (0,1,0) |  |  | -4.50 | < .01 | -16.28 | 18.31 | < .01 |
| (0,1,1)^a^ |  |  |  |  | -25.39 | 4.15 | .53 |
| (0,1,0) (0,1,0) |  |  |  |  | -16.09 | 9.15 | .17 |
| (0,1,0) (0,1,1) |  |  |  |  | -21.67 | 6.18 | .29 |
| (0,1,0) (1,1,0) |  |  |  |  | -19.22 | 5.32 | .38 |
| October 2020 |  |  |  |  |  |  |  |
| (0,1,0) |  |  |  |  | -16.62 | 17.52 | < .01 |
| (0,1,1)^a^ |  |  |  |  | -25.96 | 3.37 | .64 |
| (0,1,0) (0,1,0) |  |  |  |  | -13.37 | 13.81 | .03 |
| (0,1,0) (1,1,0) |  |  |  |  | -18.34 | 6.07 | .30 |
| (0,1,0) (0,1,1) |  |  |  |  | -20.20 | 7.58 | .18 |
| Unique Female Patients | | | | | | | |
| March 2020 |  |  |  |  |  |  |  |
| (0,0,0) | .03 | .86 | -2.38 | .42 |  |  |  |
| (0,1,0) |  |  | -3.95 | .02 | -8.78 | 20.21 | < .01 |
| (0,1,1)^a^ |  |  |  |  | -14.67 | 7.73 | .17 |
| (0,1,0) (0,1,0) |  |  |  |  | 1.81 | 4.13 | .66 |
| (0,1,0) (0,1,1) |  |  |  |  | 2.30 | 8.18 | .15 |
| October 2020 |  |  |  |  |  |  |  |
| (0,1,0) |  |  |  |  | -9.64 | 21.31 | < .01 |
| (0,1,1)^a^ |  |  |  |  | -21.17 | 6.59 | .25 |
| (0,1,0) (0,1,0) |  |  |  |  | 1.48 | 12.87 | .05 |
| (0,1,0) (1,1,0) |  |  |  |  | 1.99 | 15.37 | < .01 |
| (0,1,0) (0,1,1) |  |  |  |  | 1.53 | 17.73 | < .01 |

*Note*. Each outcome variable was log transformed prior to ARIMA model fitting. Significant Breusch-Pagan (BP) *p*-values demark heteroscedasticity. Significant Augmented Dickey-Fuller (DF) *p*-values demark stationarity. Significant Ljung-Box (Q*) *p*-values demark the presence of autocorrelation. Models were selected based on the satisfaction of assumptions and lowest Akaike information criterion (AIC).

^a^chosen model
